# Rational Design of Ag/ZnO Hybrid Nanoparticles on Sericin/Agarose Composite Film for Enhanced Antimicrobial Applications

**DOI:** 10.3390/ijms22010105

**Published:** 2020-12-24

**Authors:** Wanting Li, Zixuan Huang, Rui Cai, Wan Yang, Huawei He, Yejing Wang

**Affiliations:** 1 State Key Laboratory of Silkworm Genome Biology, Biological Science Research Center, Southwest University, Beibei, Chongqing 400715, China; lwt260@email.swu.edu.cn (W.L.); zixuan666@email.swu.edu.cn (Z.H.); cairui0330@email.swu.edu.cn (R.C.); yw961121@email.swu.edu.cn (W.Y.); hehuawei@swu.edu.cn (H.H.); 2 Chongqing Key Laboratory of Sericultural Science, Chongqing Engineering and Technology Research Center for Novel Silk Materials, Southwest University, Beibei, Chongqing 400715, China; 3 Chongqing Key Laboratory of Soft-Matter Material Chemistry and Function Manufacturing, Chongqing 400715, China

**Keywords:** sericin, AgNPs, ZnO, antimicrobial activity, green synthesis

## Abstract

Silver-based hybrid nanomaterials are receiving increasing attention as potential alternatives for traditional antimicrobial agents. Here, we proposed a simple and eco-friendly strategy to efficiently assemble zinc oxide nanoparticles (ZnO) and silver nanoparticles (AgNPs) on sericin-agarose composite film to impart superior antimicrobial activity. Based on a layer-by-layer self-assembly strategy, AgNPs and ZnO were immobilized on sericin-agarose films using the adhesion property of polydopamine. Scanning electron microscopy, energy-dispersive X-ray spectroscopy, and X-ray powder diffraction spectroscopy were used to show the morphology of AgNPs and ZnO on the surface of the composite film and analyze the composition and structure of AgNPs and ZnO, respectively. Water contact angle, swelling ratio, and mechanical property were determined to characterize the hydrophilicity, water absorption ability, and mechanical properties of the composite films. In addition, the antibacterial activity of the composite film was evaluated against Gram-positive and Gram-negative bacteria. The results showed that the composite film not only has desirable hydrophilicity, high water absorption ability, and favorable mechanical properties but also exhibits excellent antimicrobial activity against both Gram-positive and Gram-negative bacteria. It has shown great potential as a novel antimicrobial biomaterial for wound dressing, artificial skin, and tissue engineering.

## 1. Introduction

Sericin is a natural hydrophilic macromolecular protein produced by the silkworm, *Bombyx mori* [1,2]. Although sericin is a promising biomaterial for its good reactivity, hydrophilicity, biodegradability, and biocompatibility [3], its poor mechanical performance due to the amorphous structure restricts the biomaterial-related applications. Sericin has high levels of serine and aspartic acid. The polar side groups such as hydroxyl, carboxyl, and amino groups make it easy to interact with other polymers through blending, cross-linking, or copolymerization to yield improved biomaterials [1,4,5,6]. Agarose is a neutral polysaccharide derived from several species of red marine algae. It has a linear structure, consisting of D-galactose and 3,6-anhydrogalactose through β-1,4 and α-1,3 to alternately form repeating disaccharide units. It can form strong gels at low concentrations [7,8,9]. Considering its malleable mechanical properties, low cost, and good biocompatibility, agarose is often used to improve the performance of sericin as an auxiliary biomaterial [9,10,11,12].

The emergence of multidrug-resistant bacteria caused by the misuse of antibiotics has become a serious global health problem [13]. Considering its potential health risks and the incidence of cross-contamination, developing novel and effective fungicides is one of the most concerning issues worldwide [14,15,16]. Silver nanoparticles (AgNPs) are one of the most attractive inorganic antibacterial materials. It has exhibited excellent antimicrobial activity against a variety of microorganisms [17,18,19,20,21]. The antimicrobial activity is significantly affected by AgNPs size. The smaller the particles, the higher the antimicrobial efficiency [22,23,24]. However, with the decrease in particle size, AgNPs tend to aggregate, which decreases their antibacterial properties. Recently, AgNPs are incorporated in various materials such as TiO_2_, SiO_2_, ZnO, Fe_2_O_3_, and graphene, which have improved their dispersibility with enhanced antibacterial properties and long-term stability [25,26,27,28,29]. As an economic and non-toxic compound, zinc oxide nanoparticle (ZnO) is mostly used in cosmetics and biomedical materials. It can effectively prevent AgNPs aggregation with its good dispersion. The potential cytotoxicity of excessive AgNPs has attracted great attention. To ensure the biosafety and efficiency of AgNPs, incorporating metal oxides such as ZnO in AgNPs is an effective way to reduce the potential cytotoxicity of AgNPs without compromising their antimicrobial property [15,29,30]. In addition, AgNPs/ZnO hybrids have exhibited a synergistic antibacterial effect to inhibit bacterial growth or kill bacteria due to the strong interaction between AgNPs and ZnO [29,31,32,33].

Inspired by mussel secretory proteins, polydopamine (PDA) is produced by the self-polymerization of dopamine (3,4-dihydroxy-phenylalanine, DA) and has been widely applied as an effective adhesive agent [34,35]. PDA could effectively improve the adhesion ability and biocompatibility of materials. More importantly, it can promote the formation of metal nanoparticles with its reducing ability of the catechol group [35,36,37,38].

The purpose of this study is to develop sericin-based biomaterials loaded with AgNPs/ZnO for enhanced antimicrobial applications. Herein, agarose was blended with sericin to yield sericin/agarose (SS/AG, M) composite film with enhanced mechanical properties. Inspired by the adhesion and reduction properties of PDA, PDA was then coated on the surface of SS/AG film to capture ZnO and silver ions (Ag^+^) one by one, respectively, yielding PDA-SS/AG (MP), ZnO-PDA-SS/AG (MPZ), AgNPs-PDA-SS/AG (MPA), and AgNPs-PDA-ZnO-PDA-SS/AG (MPAZ) composite films after Ag^+^ was reduced to AgNPs by PDA. The morphology, structure, and properties of the films were well characterized, and the antimicrobial ability of these films against Gram-positive and Gram-negative bacteria was evaluated. This novel Ag@ZnO composite film has exhibited great potentials as a promising biomaterial for enhanced antimicrobial applications.

## 2. Results

**Figure 1:**
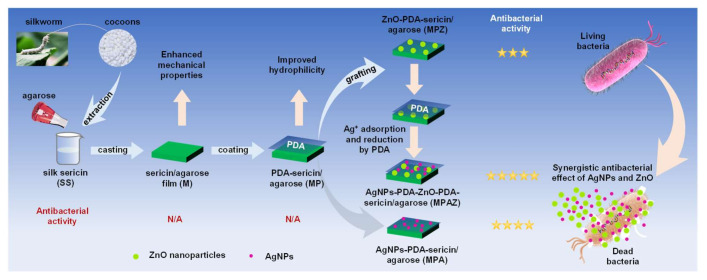
Preparation of AgNPs-PDA-ZnO-PDA-SS/AG (MPAZ) film with enhanced mechanical performance and antibacterial activity.

To overcome the inherent brittleness of sericin and improve its antibacterial activity, we developed a simple, environment-friendly strategy to enhance the performance of sericin and expand its application in biomaterials. With the aid of agarose, we prepared a composite film by blending sericin and agarose, which greatly improved the mechanical properties of sericin. PDA was coated on the surface of the composite film to assist the assembly of ZnO and AgNPs layer by layer. Silver ions were adsorbed by PDA and then reduced to AgNPs by the catechol group of PDA [38,39,40]. The presence of ZnO not only improved the dispersibility and stability of AgNPs but also enhanced the antibacterial activity of AgNPs through a synergistic effect. The preparation and antibacterial effect of the films were shown in Figure 1.

### 2.1. SEM, EDX and XRD

**Figure 2:**
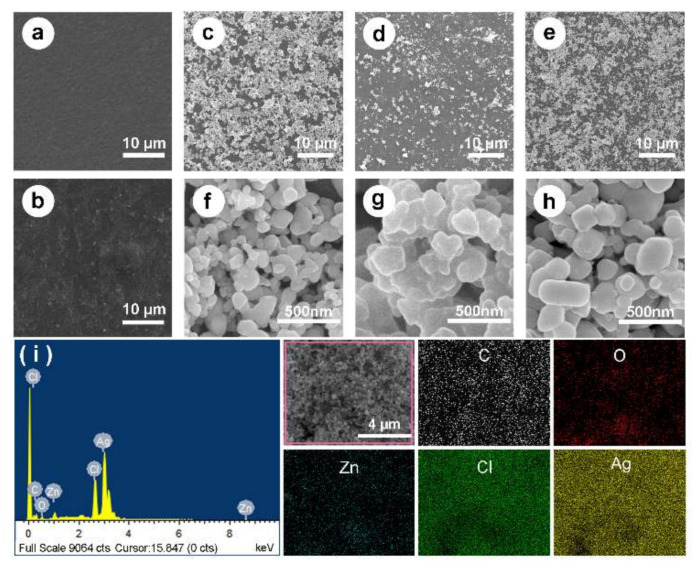
SEM and EDX. (**a**–**h**) The morphologies of different films are characterized by SEM. (**a**), M; (**b**), MP; (**c**–**e**), MPZ, MPA, and MPAZ, respectively; (**f**–**h**), higher magnification images of MPZ, MPA, and MPAZ, respectively; (**i**), EDX analysis of the elemental mapping of the selected region (indicated by a pink box) revealed the presence of zinc (Zn, blue), carbon (C, white), oxygen (O, red), silver (Ag, yellow) and chlorine (Cl, green), and the uniform distribution of ZnO and AgNPs on the surface of MPAZ film.

Figure 2 presented the surface morphologies of M, MP, MPZ, MPA, and MPAZ films by scanning electron microscope (SEM) under different magnification. Sericin itself could not form a film due to its natural brittleness. SS/AG film (M) had a smooth surface (Figure 2a). After dopamine modification, MP exhibited a rough surface morphology (Figure 2b). With the addition of AgNPs and/or ZnO, some irregular particles appeared on the surface of the composite (Figure 2c–h). Higher magnification images showed the aggregation of AgNPs on the surface of the MPA film (Figure 2g), while AgNPs were relatively dispersed on the surface of MPAZ film due to the presence of ZnO (Figure 2h).

The composition of the MPAZ film was further characterized by energy-dispersive X-ray spectroscopy (EDX), as shown in Figure 2i. The elemental mapping of the selected region (indicated by a pink box) revealed the presence of zinc (Zn, cyan), carbon (C, white), oxygen (O, red), silver (Ag, yellow), and chlorine (Cl, green), and the uniform distribution of ZnO and AgNPs on the surface of MPAZ film. The characteristic peak of silver was at ~2.9 kV. The results further demonstrated that Ag^+^ was effectively reduced to AgNPs by PDA.

**Figure 3:**
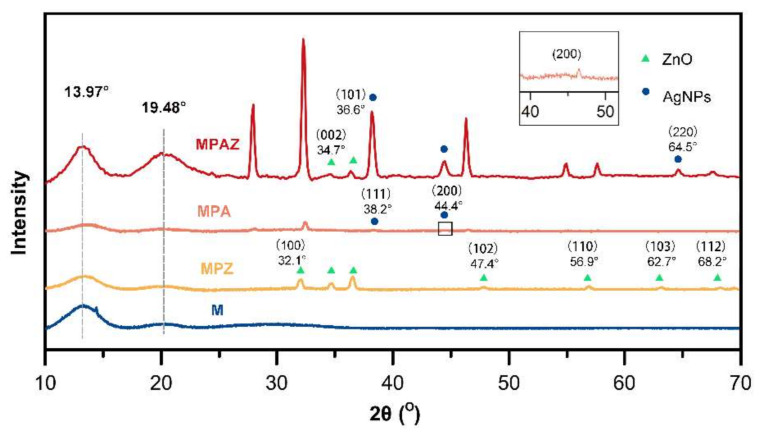
The characteristic XRD patterns of the films.

To further identify the crystalline structure of ZnO and AgNPs, X-ray powder diffraction (XRD) was performed. All films showed the characteristic patterns of sericin and agarose (Figure 3), which appeared at 19.48° and 13.97° [41,42], respectively. The diffraction patterns of ZnO on the MPZ film could be assigned to the (100), (002), (101), (102), (110), (103), and (112) crystalline structure of ZnO (Figure 3) according to the JCPDS card of ZnO (No. 00-36-1451), which are located at 32.1°, 34.7°, 36.6°, 47.4°, 56.9°, 62.7°, and 68.2°, respectively. Two characteristic diffraction patterns were observed at 38.2° and 44.4° on the MPA film (Figure 3), corresponding to the (111) and (200) planes of AgNPs (JCPDS card No. 00-004-0783), respectively [43,44,45]. The XRD of MPAZ showed the specific patterns corresponding to the crystalline structure of ZnO and AgNPs [46,47,48], indicating that ZnO and AgNPs were successfully coated on the MPAZ film. The XRD pattern of ZnO did not change after AgNPs loading, indicating that AgNPs were not incorporated into the lattice of ZnO, but only deposited on the surface of ZnO [47]. The characteristic pattern (100) of ZnO was not observed on the MPAZ film, which may overlap with the pattern of Ag_2_O (2θ = 32.2°) as they are very close to each other [49]. Ag_2_O may be derived from the oxidation of AgNPs on the surface of the film exposed to the air in aqueous solutions [50]. The combination of AgNPs and ZnO makes the crystal larger, which may lead to an increase in the diffraction intensity of ZnO on the MPAZ film. Meanwhile, the dispersion of ZnO resulted in an increase in the diffraction intensity of AgNPs on the MPAZ film [51,52].

### 2.2. Swelling Ratio and Water Contact Angle

**Figure 4:**
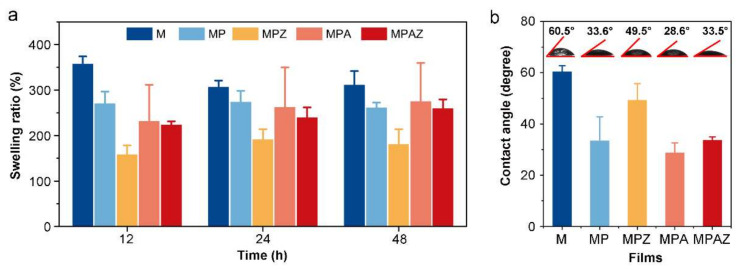
Swelling ratio and water contact angle of different films. a, Swelling ratio of the films; b, Water contact angle of the films.

The swelling ratio is a feasible method to value the water absorption ability of materials. Figure 4a showed the swelling ratio of each film. The swelling ratio of SS/AG film was 382.73% at 12 h. The swelling ratio of the composite film coated with PDA and metal nanoparticles ranged from 157.93% to 269.84%. After two days, the swelling ratio of SS/AG film was almost unchanged, while the swelling ratios of other films slightly increased, which may be ascribed to the water absorption capability of PDA. The results suggested that composite films have excellent water absorption capability.

The water contact angle indicates the hydrophilicity of materials. Generally speaking, the smaller the contact angle, the better the hydrophilicity of a material [53]. The water contact angle of M, MP, MPZ, MPA, and MPAZ was 60.5°, 33.6°, 49.5°, 28.6°, and 33.5° (Figure 4b), respectively. All water contact angles were less than 90°, indicating the films were hydrophilic. The water contact angle decreased after AgNPs modification compared to that of MP, while the water contact angle increased after ZnO modification, which may be due to the difference of the hydrophilicity of AgNPs and ZnO [54,55].

### 2.3. Mechanical Properties

**Figure 5:**
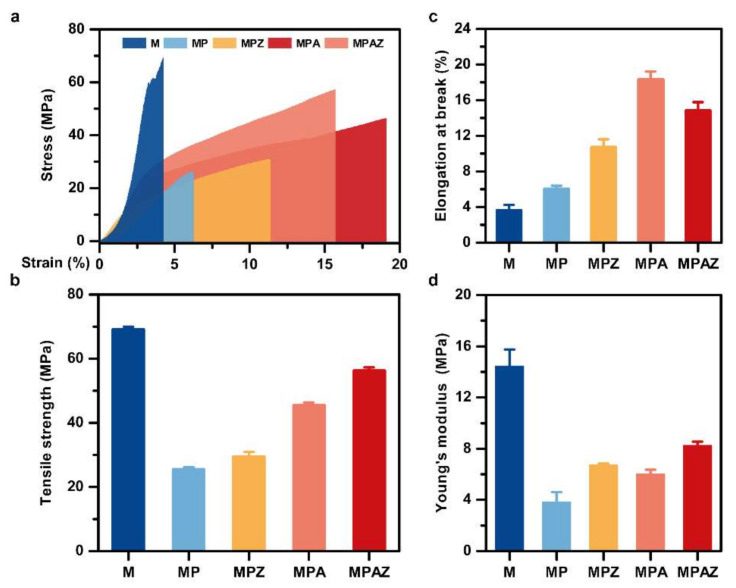
Mechanical properties of different films. (**a**), Stress-strain curves; (**b**), tensile strength; (**c**), elongation at break; (**d**), Young’s modulus.

The molecular spatial structure of sericin was disordered, resulting in a lack of mechanical properties. Here, agarose was blended with sericin to improve its mechanical properties. A stereomicroscope was used to determine the thickness of different films (Figure S1). The statistical results from five independent tests were shown in Table S1. Figure 5 presented the stress-strain curves and Young’s modulus of the films. The results showed that the mechanical properties of sericin had been greatly improved after the incorporation of agarose. As shown in Figure 5a–c, M had the tensile strength of 70.4 MPa, which was the highest among all films. However, its elongation at break (strain) was really the weakest, at only 4.3%. However, with the addition of metal nanoparticles, the thickness of the film increased, resulting in greatly improved mechanical properties of the film. Taking into account both the stress and strain, the mechanical properties of MPAZ were the best. It had a tensile stress of 57.37 MPa and a strain of 15.70%, respectively. Young’s modulus also indicated the same result (Figure 5d). In general, the addition of ZnO and AgNPs improved the mechanical properties of the MPAZ film, which can meet the requirements of biomaterials to a certain extent [56,57,58].

### 2.4. Inhibition Zone Assay

**Figure 6:**
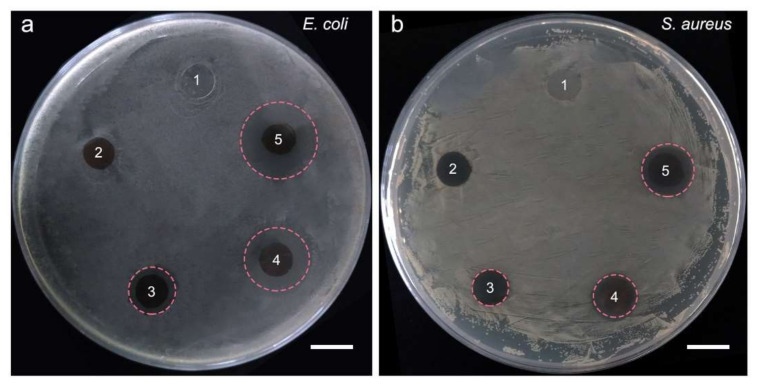
Inhibition zone assay of different films. (**a**), *E. coli*; (**b**), *S. aureus*. 1–5, M, MP, MPZ, MPA, and MPAZ, respectively. Scale bar, 1 cm.

*Escherichia coli* (*E. coli*) and *Staphylococcus aureus* (*S. aureus*) are typical Gram-negative and Gram-positive bacteria, respectively, which were used to evaluate the antibacterial properties of the films in this study. It was clear that M and MP did not form visible inhibition zones against *E. coli* or *S. aureus*. However, after the modification of ZnO or/and AgNPs, the films had formed obvious inhibition zones, both against *E. coli* and *S. aureus* (Figure 6), indicating the antibacterial activities of the films.

The diameters of the bacteriostatic zones were listed in Table 1. According to the diameters of the bacteriostatic zones, the antibacterial activity was ranked in the order of MPAZ, MPA, and MPZ. M and MP had no obvious antibacterial effect.

### 2.5. Colony Counting Assay

**Figure 7:**
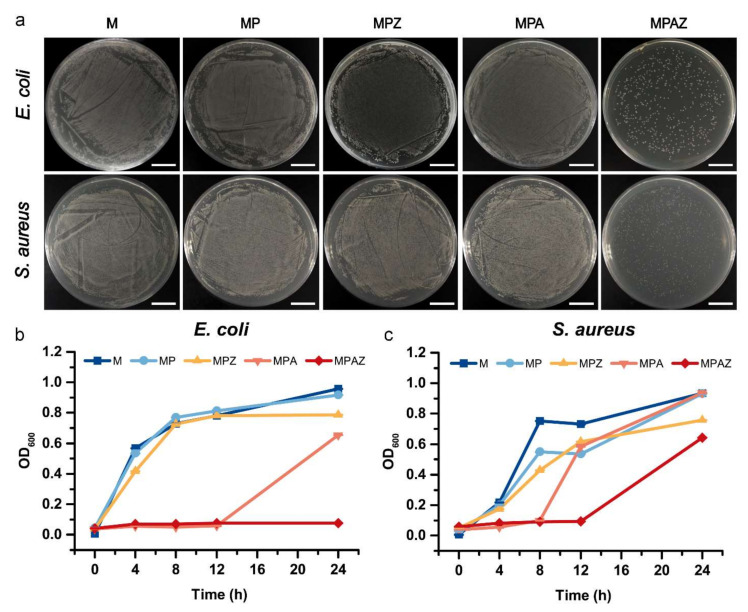
Colony counting and bacterial growth curve assays. (**a**), The colonies of *E. coli* and *S. aureus* after culture in the presence of different films for 24 h and 12 h, respectively; (**b**,**c**), bacterial growth curves in the presence of different films. Scale bar, 1 cm.

After culture in the presence of different films for 24 h and 12 h, *E. coli* and *S. aureus* were spread on the plates to value the antibacterial activity of the films by counting the formed colony numbers, respectively, as shown in Figure 7a. Compared with other groups, the colony number in the MPAZ group were greatly reduced, both for *E. coli* and *S. aureus*, indicating that MPAZ had the best antibacterial effect among all tested films.

### 2.6. Growth Curve Assay

A bacterial growth curve assay was performed to compare the antibacterial effect of the films further. The result showed that M and MP did not affect bacterial growth. MPZ slightly repressed the bacterial growth compared with M and MP. Noticeably, both MPA and MPAZ effectively inhibited the growth of *E. coli* and *S. aureus*. In the presence of MPA, the growth of *E. coli* and *S. aureus* were delayed by 12 h and 8 h, respectively. However, in the presence of MPAZ, the growth of *E. coli* and *S. aureus* were arrested for 24 h and 12 h, respectively (Figure 7b,c). It was noted that after 24 h and 12 h, the OD of *E. coli* and *S. aureus* in the MPZ and MPA groups were almost comparable to those in the M and MP groups, respectively. Hence, the number of colonies on the plates seemed to be indistinguishable among MPZ, MPA, M, and MP groups (Figure 7a). The result was consistent with the above results, suggesting the antibacterial effect of MPAZ was the best among all films.

### 2.7. LIVE/DEAD BacLight Cell Viability Assay

**Figure 8:**
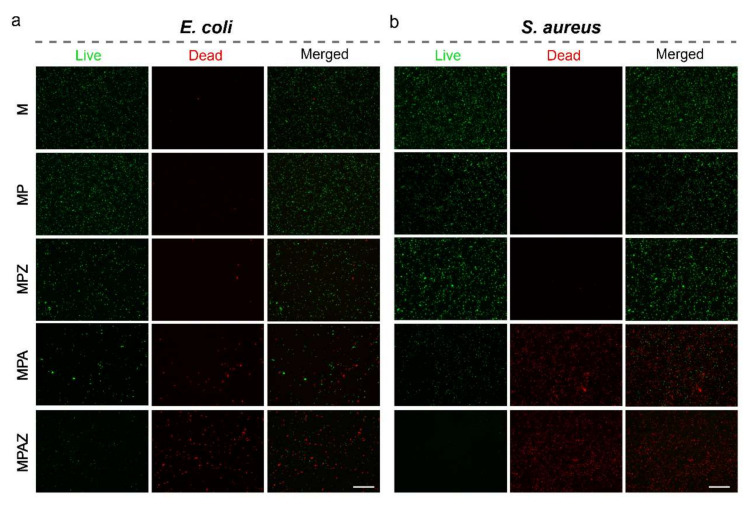
LIVE/DEAD BacLight cell viability assay. Fluorescence staining of *E. coli* (**a**) and *S. aureus* (**b**) cells after culture in the presence of different films. Scale bar, 50 μm.

Further, a LIVE/DEAD BacLight cell viability assay was performed to visualize the bactericidal capability of the films. In this assay, living cells are stained green, while dead cells are stained red [59,60]. The results showed that most bacteria were stained red in the presence of the MPA or MPAZ film, while most bacteria were dyed green in the presence of the M, MP, or MPZ film (Figure 8). The number of dead cells (red) indicated that the bactericidal activity of MPAZ was better than that of other films.

### 2.8. SEM of Bacteria

**Figure 9:**
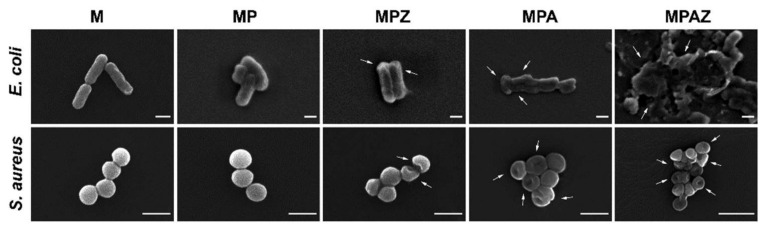
SEM of bacteria in the presence of different films. The arrows indicated the deformation, shrinking, and damage of bacterial cell walls. Scale bar, 1 μm.

Further, SEM was performed to visualize the interaction of MPAZ with the bacterial cells. In the presence of M or MP film, *E. coli* remained in their state in solution with intact cell walls. However, in the presence of MPZ, some bacterial cell walls remained intact but underwent deformation, indicating they were in a poor living state. MPA resulted in the significant shrink of *E. coli* cell walls under high magnification. In the presence of MPAZ, the bacterial cell wall suffered severe damage, resulting in cytoplasmic leakage (Figure 9). Similarly, *S. aureus* was spherical with a smooth and intact cell wall in the presence of M or MP film, but MPZ caused a small number of cell walls to shrink, and more cells with shrinking walls were found in the presence of MPA. MPAZ not only caused a large number of bacterial walls to shrink but also induced the deformation of cell walls (Figure 9). The results suggested that the bactericidal mechanism of MPAZ was partly due to the disruption of bacterial cell wall integrity and the resultant bacterial cells lysis, in which ZnO could promote the generation of reactive oxygen species and destroy the structure of cell membranes, and AgNPs could kill bacteria by releasing silver ions to destroy bacterial cell walls [61,62].

*S. aureus* appeared to be more resistant to MPAZ than *E. coli*, which may be due to its special structure. The cell wall of *S. aureus* was significantly different from that of *E. coli*. The content of peptidoglycan in the cell wall of *S. aureus* is higher than that of *E. coli*, increasing the thickness of the cell wall, which helps *S. aureus* to protect its cells from the penetration of silver ions/AgNPs/ZnO into the cytoplasm [35]. In addition, our results suggested that MPAZ had the best antibacterial activity among all films, which may be due to the synergistic antibacterial effect of AgNPs and ZnO [15,27,30].

## 3. Materials and Methods

### 3.1. Materials and Chemicals

Silkworm cocoons were kindly provided by the State Key Laboratory of Silkworm Genome Biology, Southwest University (Beibei, Chongqing, China). Silver nitrate (AgNO_3_), ZnO, and dopamine hydrochloride were from Aladdin (Shanghai, China). Agarose was provided by Biowest (Shanghai, China). Tris (hydroxymethyl) aminomethane (Tris) was from Sangon Biotech (Shanghai, China). *E. coli* (CICC 10389) and *S. aureus* (CICC 21600) were from the China Center of Industrial Culture Collection. LIVE/DEAD BacLight bacterial viability kit (L34856) was purchased from Thermo Fisher Scientific (Waltham, MA, USA). All other chemicals were of analytical grade and used directly.

### 3.2. Preparation of the Composite Films

The MPAZ film was prepared as per previous reports with slight modifications [38,63]. Agarose solution (2%, *w*/*v*) was adequately mixed with sericin solution (1%, *w*/*v*) as a volume ratio of 1:1 at 65 °C. Then, the mixture was dried at 37 °C overnight to become a SS/AG film. Dopamine (2 mg) was dissolved in 10 mL Tris-HCl, pH 8.5, and used immediately. Next, the SS/AG film was soaked in fresh dopamine solution at 25 °C for 6 h. After washing 3 times with water, the SS/AG film was dried at 25 °C for 12 h to yield a PDA-coated SS/AG (MP) film. Then, the MP films were immersed into ZnO solution (0.01 M) and AgNO_3_ solutions (0.01 M) with shaking (50 rpm) at 25 °C for 2 h, respectively. After washing 3 times with water, the films were dried at 25 °C for 12 h to obtain ZnO-PDA-SS/AG (MPZ) film and AgNPs-PDA-SS/AG (MPA) film, respectively. The MPZ film was soaked into fresh dopamine solution at 25 °C for 15 min to coat PDA on its surface and then immersed into AgNO_3_ solution (0.01 M) at 25 °C for 2 h after washing 3 times with water. After 3 rinses with water, the film was dried at 25 °C to yield AgNPs-PDA-ZnO-PDA-SS/AG film (MPAZ).

### 3.3. SEM, EDX, and XRD

The morphologies of different films were characterized by a field emission scanning electron microscope HITACHI SU8010 (Tokyo, Japan). Prior to SEM observation, the films with a dimension of 1 cm width and 1 cm height were precoated with Au for 90 s and then imaged on SU8010 with 5~10 kV acceleration voltage. The element mapping of MPAZ film was characterized by SU8010 equipped with an energy-dispersive X-ray spectroscopy mapping system at 15 kV. XRD spectra were recorded on a PANalytical x’pert (Almelo, The Netherlands) within 10–70° at a speed of 2°/min to identify the special crystalline structure of the composites.

### 3.4. Hydrophilicity and Swelling Ratio

The hydrophilicity of the film was measured by the sessile drip contact angle using a KRÜSS DSA100 contact angle analyzer (Hamburg, Germany) at 25 °C. The water contact angle was measured by dropping 4 μL water on the surface of the film at a time. Each sample was measured in triplicate. The result was an average of 3 tests.

The swelling ratio was used to characterize the water absorption capacity of the films. The dry films (1.5 cm × 1.5 cm, length × width) were immersed in water. Then, the films were removed out at different intervals and weighed after wiping off excess water with clean paper. The swelling ratio was defined as follows:

Swelling ratio (%) = (m2 − m1)/m1 × 100%(1)

where m1 and m2 were the mass of dry and swollen films, respectively. Three replications were performed for each film to ensure the accuracy of the test.

### 3.5. Mechanical Properties

The tensile strength (stress) and elongation at break (strain) of the films were measured on SHIMADZU AG-X plus (Tokyo, Japan). The thickness of the film was measured individually on a Stemi 2000C stereomicroscope (Shanghai, China), and the average value of 5 independent tests were applied. The samples (4 cm × 1 cm, length × width) were fixed on the mold and then stretched at a speed of 10 mm/min. The stress and strain values were recorded in real-time during operation. Each film was examined for at least 5 replicates to ensure the accuracy of the test, and the average value was calculated. Young’s modulus was determined from the corresponding stress–strain curve.

### 3.6. Inhibition Zone Assay

*E. coli* and *S. aureus* were used to evaluate the antibacterial activity of the films. The bacteria were inoculated into Luria-bertani (LB) medium, and then cultured at 37 °C for 12 h with 220 rpm shaking speed until the optical density value at 600 nm (OD600) reached 1.0. Then, the bacterial suspension (200 μL) was collected and uniformly spread on an agarose plate. Next, the circular films (diameter, 0.7 cm) were sterilized by ultraviolet and then placed on the surface of the plate. After 12 h of incubation at 37 °C, the inhibition zones around the films were photographed, and the diameters were measured.

### 3.7. Colony Counting and Growth Curve Assays

The antibacterial properties of the films were further evaluated by colony counting and growth curve assays. Bacteria (1 × 10^7^−10^8^ colony-forming unit (CFU)/mL) were inoculated into LB medium and cultured at 37 °C in the presence of different films. Then, bacterial suspension (0.4 mL) was collected at different intervals to determine the bacterial growth curve by measuring the OD600 of the bacteria. *E. coli* and *S. aureus* were cultured in the presence of the films for 24 h and 12 h, respectively, and then spread on the plates after 500 times dilution with the media. The plates were photographed to compare the number of active bacterial colonies on the plates after culture at 37 °C for 12 h.

### 3.8. LIVE/DEAD BacLight Cell Viability Assay

The bacteria (10^9^ CFU/mL) were cultured in LB medium in the presence of different films at 37 °C for 4 h. After removal of the films, the bacteria were collected by centrifugation (5000 rpm, 10 min), rinsed twice with phosphate-buffered saline (PBS, pH 7.4). Next, the bacteria were suspended in 100 μL PBS buffer (pH 7.4) and mixed with 10 μL staining reagent. After 15 min of incubation in the dark, as per the LIVE/DEAD BacLight cell viability guide kit, the living/dead bacteria cells were directly observed on a fluorescence microscopy EVOS FL auto cell imaging system (Waltham, MA, USA).

### 3.9. SEM of Bacteria

The bacteria were fixed in 2.5% glutaraldehyde after 4 h of culture in the presence of different films, rinsed twice with PBS buffer (pH 7.4), and then dehydrated by gradient ethanol concentration (30% for 15 min, 50% for 15 min, 70% for 15 min, 90% for 15 min, and 100% for 15 min). Finally, the morphologies of the bacteria in the presence of different films were characterized by SEM after air-drying overnight.

## 4. Conclusions

In summary, we developed a simple and eco-friendly strategy to efficiently assemble ZnO and AgNPs on sericin-agarose composite film with the assistance of PDA by layer-by-layer self-assembly. The resultant MPAZ film has not only desirable hydrophilicity, high water absorption ability, and favorable mechanical properties but also exhibits excellent antimicrobial activity against both Gram-positive and Gram-negative bacteria. Therefore, it is promising as a novel antimicrobial material for the applications such as antibacterial coatings, wound dressing, and tissue engineering.

## Figures and Tables

**Table 1:** The diameters of the bacteriostatic zones of different films.

	M (cm)	MP (cm)	MPZ (cm)	MPA (cm)	MPAZ (cm)
*E. coli*	0 ± 0.02	0 ± 0.02	0.25 ± 0.05	0.93 ± 0.03	1.05 ± 0.05
*S. aureus*	0 ± 0.02	0 ± 0.02	0.04 ± 0.01	0.34 ± 0.04	0.45 ± 0.05

## Supplementary Materials

The following are available online at https://www.mdpi.com/1422-0067/22/1/105/s1, Figure S1: Microscopic pictures of cross-sections of M (a), MP (b), MPZ (c), MPA (d), MPAZ (e) composite films, Table S1: Thickness of different films.
