# Supplementary material for: Rational Design of Ag/ZnO Hybrid Nanoparticles on Sericin/Agarose Composite Film for Enhanced Antimicrobial Applications
Source: Int J Mol Sci. 2020 Dec 24;22(1):105. doi: 10.3390/ijms22010105 (PMC7794692; doi:10.3390/ijms22010105)
Supplement: Supplementary file 1 [file ijms-22-00105-s001.pdf]

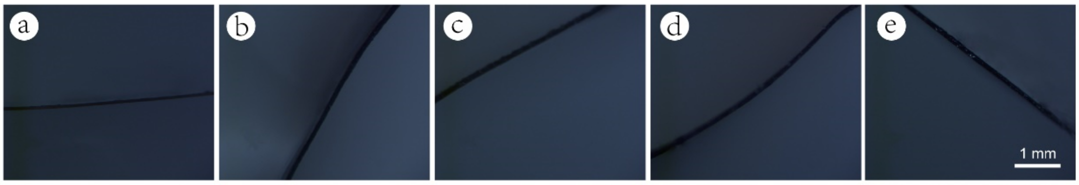

Figure S1. Microscopic pictures of cross-sections of M (a), MP (b), MPZ (c), MPA (d), MPAZ (e) composite films.

Table 1. Thickness of different films.

Film	M	MP	MPZ	MPA	MPAZ
Thickness (μm)	28.82 ± 0.18	36.50 ± 0.75	47.97 ± 5.05	50.23 ± 2.20	58.47 ± 2.20
